# Association of Female Menopause With Atrioventricular Mechanics and Outcomes

**DOI:** 10.3389/fcvm.2022.804336

**Published:** 2022-04-21

**Authors:** Kuo-Tzu Sung, Chanchal Chandramouli, Chi-In Lo, Jui-Peng Tsai, Yau-Huei Lai, Chih-Chung Hsiao, Shin-Yi Tsai, Chun-Ho Yun, Ta-Chuan Hung, Jen-Yuan Kuo, Jiun-Lu Lin, Charles Jia-Yin Hou, Ying-Ju Chen, Cheng-Huang Su, Chung-Lieh Hung, Bernard E. Bulwer, Hung-I Yeh, Carolyn S. P. Lam

**Affiliations:** ^1^Division of Cardiology, Department of Internal Medicine, MacKay Memorial Hospital, Taipei, Taiwan; ^2^Department of Medicine, Mackay Medical College, New Taipei City, Taiwan; ^3^Institute of Clinical Medicine, National Yang Ming University, Taipei, Taiwan; ^4^National Heart Research Institute, National Heart Centre Singapore, Singapore, Singapore; ^5^Academic Clinical Programme, Duke-National University of Singapore, Singapore, Singapore; ^6^Department of Biomedical Imaging and Radiological Science, National Yang Ming University, Taipei, Taiwan; ^7^School of Public Health, Johns Hopkins University Bloomberg, Baltimore, MD, United States; ^8^Department of Telehealth, MacKay Memorial Hospital, Taipei, Taiwan; ^9^Institute of Biomedical Sciences, Mackay Medical College, New Taipei City, Taiwan; ^10^Brigham and Women’s Hospital, Boston, MA, United States; ^11^Massachusetts College of Pharmacy and Health Sciences, Boston, MA, United States; ^12^Department of Medicine, University Medical Centre Groningen, Groningen, Netherlands

**Keywords:** women, echocardiography, remodeling, heart failure, left atrial strain, menopause, estrogen

## Abstract

**Background:**

Despite known sex differences in cardiac structure and function, little is known about how menopause and estrogen associate with atrioventricular mechanics and outcomes.

**Objective:**

To study how, sex differences, loss of estrogen in menopause and duration of menopause, relate to atrioventricular mechanics and outcomes.

**Methods:**

Among 4051 asymptomatic adults (49.8 ± 10.8 years, 35%women), left ventricular (LV) and left atrial (LA) mechanics were assessed using speckle-tracking.

**Results:**

Post-menopausal (vs. pre-menopausal) women had similar LV ejection fraction but reduced GLS, reduced PALS, increased LA stiffness, higher LV sphericity and LV torsion (all *p* < 0.001). Multivariable analysis showed menopause to be associated with greater LV sphericity (0.02, 95%CI 0.01, 0.03), higher indexed LV mass (LVMi), lower mitral e’, lower LV GLS (0.37, 95%CI 0.04–0.70), higher LV torsion, larger LA volume, worse PALS (∼2.4-fold) and greater LA stiffness (0.028, 95%CI 0.01–0.05). Increasing years of menopause was associated with further reduction in GLS, markedly worse LA mechanics despite greater LV sphericity and higher torsion. Lower estradiol levels correlated with more impaired LV diastolic function, impaired LV GLS, greater LA stiffness, and increased LV sphericity and LV torsion (all *p* < 0.05). Approximately 5.5% (37/669) of post-menopausal women incident HF over 2.9 years of follow-up. Greater LV sphericity [adjusted hazard ratio (aHR) 1.04, 95%CI 1.00–1.07], impaired GLS (aHR 0.87, 95%CI 0.78–0.97), reduced peak left atrial longitudinal strain (PALS, aHR 0.94, 95%CI 0.90–0.99) and higher LA stiffness (aHR 10.5, 95%CI 1.69–64.6) were independently associated with the primary outcome of HF hospitalizations in post-menopause. Both PALS < 23% (aHR:1.32, 95%CI 1.01–3.49) and GLS < 16% (aHR:5.80, 95%CI 1.79–18.8) remained prognostic for the incidence of HF in post-menopausal women in dichotomous analyses, even after adjusting for confounders. Results were consistent with composite outcomes of HF hospitalizations and 1-year all-cause mortality as well.

**Conclusion:**

Menopause was associated with greater LV/LA remodeling and reduced LV longitudinal and LA function in women. The cardiac functional deficit with menopause and lower estradiol levels, along with their independent prognostic value post-menopause, may elucidate sex differences in heart failure further.

## Introduction

Sex differences in cardiovascular disease, particularly heart failure (HF), have been highlighted in several recent publications (1–3). Whereas sex differences in age-related cardiovascular changes have been well-described (4), little is known about how female menopause and sex hormones associate with atrioventricular mechanics and outcomes.

The risk of CVD in women accelerates at later stage in life, often coinciding with their menopause transition. Prior studies have evidenced key alterations associated with menopause, including changes in endogenous sex hormones levels, body fat distribution and cardiometabolic health (5–7). While transition into menopause is not formally recognized as a risk factor of CVD in the guidelines, there is a compelling adverse cardiometabolic changes accompanying midlife and menopause (8). Collectively, these maladaptive changes in menopause are potentially associated with deteriorating myocardial function, i.e., greater left ventricular (LV) diastolic dysfunction (4, 9, 10), increased concentric LV remodeling (9–12), and altered cardiac deformational measures (13), potentially rendering post-menopausal women susceptible to HF with preserved ejection fraction (HFpEF) (14–18). However to date, no study has comprehensively looked at the relationship between menopause, circulating estradiol levels and atrioventricular deformation measures. Previous studies were relatively small, lacked age-matched comparisons between sexes and did not involve advanced echocardiographic techniques (9–13, 18). Therefore, we investigated the association between sex, menopause and increasing years of menopause on cardiac (LA and LV) geometry and mechanics in a large asymptomatic population using chamber-specific, speckle-tracking technique (9–13, 18).

## Materials and Methods

### Study Population

**FIGURE 1:**
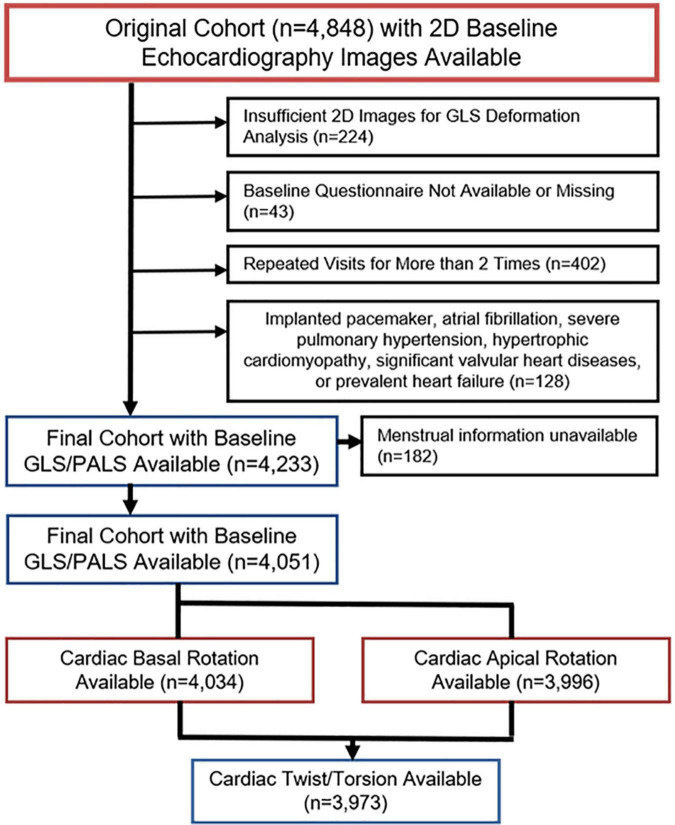
Schematic flow diagram after exclusion and final selection of study population.

We recruited 4,051 consecutive participants without prevalent HF who underwent annual cardiovascular health survey from June 2009 to December 2012 in Mackay Memorial Hospital, a tertiary medical center in Taiwan. Comprehensive data collection at baseline included physical examinations, anthropometric measures, chest radiography, body surface area, 12-leads electrocardiogram (ECG), blood test and biochemistry. A structured questionnaire was used to obtain baseline demographics, medical history, clinical symptoms, signs and lifestyles. For women, histories of gynecological surgeries, marital status, menarche status, the use of oral contraceptives and hormone therapy for menopause were also provided. Age at menarche was obtained from self-reported questionnaires or extracted from the respective institution’s electronic chart system records of obstetrics/gynecology clinic visits, where available. Menopause was defined retrospectively as the time of the final menstrual period for more than 1 year of no menstrual periods. The study details, inclusion and exclusion criteria were as published previously (19). For the purpose of this study, the following exclusion criteria were defined: Presence of hypertrophic cardiomyopathy, previous cardiac valvular surgery, atrial fibrillation or other arrhythmias (that preclude consistent cycle length for ECG-gated deformation measures), congenital heart disease and at least moderate degree of valvular heart disease. The study was approved by the local ethical committee (18MMHIS109). Among 4848 study subjects eligible during our observation period, 4,051 had sufficient quality of 2D speckle-tracking images, baseline clinical demographic and menstrual information comprised the final study population (Figure 1).

### Echocardiographic Assessment of Cardiac Structure and Function

#### Conventional Echocardiography

Standardized transthoracic echocardiography evaluations were conducted on all subjects using a commercially available ultrasound machine GE Vingmed system (Vivid i), equipped with a 2-4-MHz transducer (3S-RS) (GE Vingmed Ultrasound, Norway), by an experienced technician who was blinded to the study. All subjects were assessed for LV end-diastolic and end-systolic diameters, wall dimensions, LV/LA chamber volumes and LV ejection fraction using the biplane Simpson method. LV mass was calculated using the formula according to the guideline proposed by American Society of Echocardiography in consensus with European Association of Cardiovascular Imaging (20) and was indexed to body surface area (g/m^2^). LV sphericity was defined as the ratio of the LV short-axis diameter to LV long-axis length from LV apical 4-chamber view by 2D measurement at end-diastolic phase (21, 22).

LA emptying fraction was calculated as:


$$100\times \frac{\left(\mathrm{Maximal}\,\mathrm{LA}\,\mathrm{volume}-\mathrm{Minimal}\,\mathrm{LA}\,\mathrm{volume}\right)}{\left(\mathrm{Maximal}\,\mathrm{LA}\,\mathrm{volume}\right)}$$

We further indexed LA volume (maximal) measures to height (ht^2^.7) in current study. Mitral inflow Doppler of early/late diastolic (E/A) LV filling velocities, and deceleration time were obtained from the apical 4-chamber view. Tissue Doppler imaging-based mitral annulus contraction (s’) and relaxation velocities (e’) were determined using spectral Doppler techniques from both septal and lateral annulus with averaged values presented, with LV filling pressure estimated by E divided by e’ (E/e’). All echocardiographic measures represented the average of three consecutive heart cycles.

#### Speckle Tracking Techniques for Left Atrial/Left Ventricular Strain Assessment and Torsion

**FIGURE 2:**
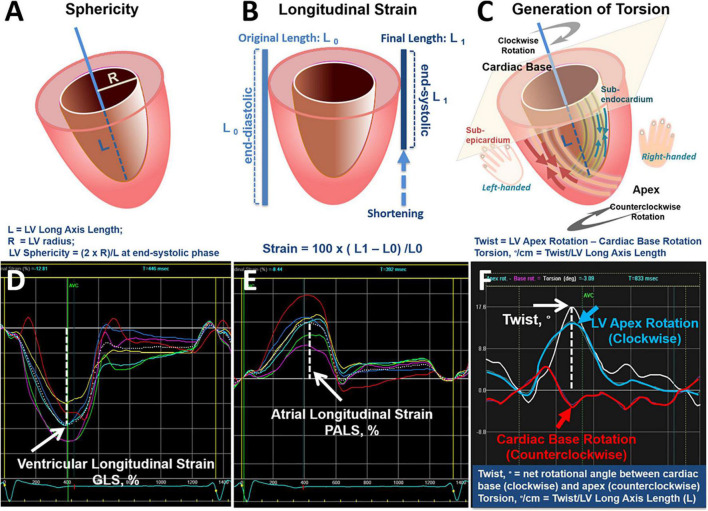
Definition of sphericity, strain, torsion and cardiac mechanics. Definition of LV sphericity **(A)** and the fraction of longitudinal systolic shortening [expressed as percentage (%)] in terms of longitudinal strain **(B)**. When the heart contracts, the apex rotates in counterclockwise direction and the base rotates in clockwise direction. Cardiac torsion as defined by the net twist angle (from opposite rotational differences between base and apex) divided by LV length (L) **(C)**. Curves from tracing of ventricular **(D)**/atrial longitudinal strain **(E)** from any single plane and calculation of twist from automatic quantification algorithm in current study were displayed **(F)**.

In brief, we used 2D speckle-tracking technique to automatically trace the region of interest, which encompasses the LV myocardial layer between the endocardial and epicardial borders. Three apical views were averaged to obtain global longitudinal strain (GLS; 2-chamber, 3-chamber and 4-chamber views) and global circumferential strain (GCS; mitral valve, papillary, LV apex short-axis layers) (23). We expressed the original negative values of GLS/GCS as absolute values | x| in this study, with greater | x| indicating better myocardial deformational measure. LA deformation was measured by manually tracing LA endocardial borders at end-systolic phase from both 4- and 2-chamer LV apical views. Peak atrial strain was defined as peak, up-sloping wave, derived from each atrial segment. Representative peak atrial longitudinal strain (PALS) was obtained by averaging both 4- and 2-chamber views for consecutive 3-beats, as described previously (24, 25). LA stiffness (^%–1^) (mechanical strain as: afterload) was derived using E/e’ divided by PALS (26). Cardiac rotation was defined as the angle produced when contraction occurs, with the left-handed subepicardial fibers and right-handed subendocardial fibers causing a clockwise and counterclockwise rotation at the base (from mitral valve as negative value) and apex from LV apex as positive value), respectively, with cardiac twist calculated as “net rotational angle” displacement between the opposing basal and apical rotations (27, 28). Torsion, a dynamic wringing motion of the left ventricle along its long axis (29), was calculated as twist divided by the long-axis (L) at end-systole (twist,°[degree])/L, degree per centimeter). Torsion-to circumferential strain (Torsion-CS) ratio was subsequently computed to eliminate the effects of LV circumferential shortening on torsional mechanics (Figure 2).

### Assessment of Circulating Estradiol

Estradiol levels were measured in serum samples of 281 (20.0%) women (average age: 47.8 ± 9.0 years) using the ARCHITECT Estradiol assay method, a Chemiluminescent Microparticle Immunoassay (CMIA) (Architect; Abbott Diagnostics, Abbott Park, IL).

### Outcomes

In the subset of post-menopausal women (*n* = 669), long-term follow-up for clinical composite outcome of HF hospitalization and all-cause mortality was available for a median of 2.9 years (IQR: 2.2∼3.9 years). Outcomes were independently adjudicated by two cardiologists reviewing clinical notes and medical records. All data were documented in an electronic database capture system, which was managed by the echocardiography core laboratory at the Mackay Memorial Hospital.

### Statistical Analysis

**FIGURE 3:**
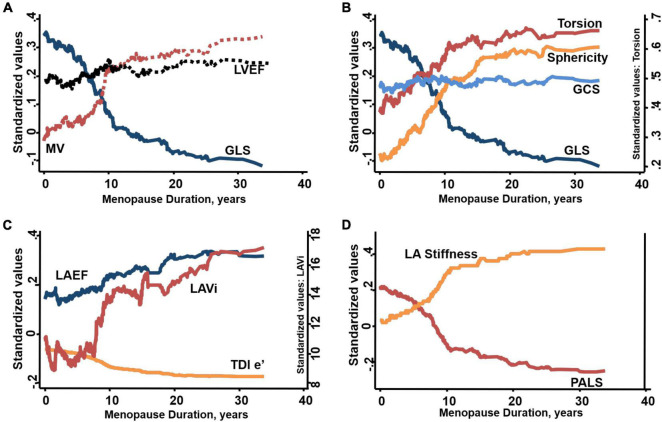
Duration of menopause-associated changes in **(A)** increased LV mass-to-volume ratio (MV) and worsened global longitudinal strain (GLS) with relatively unchanged LV ejection fraction (LVEF), **(B)** sphericity at end-diastole accompanied by greater torsion despite worsened GLS and relatively unchanged global circumferential strain (GCS), **(C)** worsened myocardial relaxation e’, greater indexed LA volume and relatively unchanged LA emptying fraction (LAEF), **(D)** worsened global peak LA strain (PALS) and greater LA stiffness in post-menopause women with time to onset of menopause available.

Data were stratified by sex, menopause status (pre- and post-menopausal) and menopause duration. Continuous variables were presented as mean ± SD and categorical variables were presented as proportions or percentages in distributions. Differences between sex, and menopause status (pre- and post-menopausal) were tested by ANOVA (Bonferroni *post hoc* test) for continuous variables and chi-square test for categorical variables (Table 1). Further, menopause status (pre- vs. post-menopause as dichotomous variable) were entered separately into multivariable models to examine their effects on cardiac structural remodeling, functional parameters (including diastolic parameters) and torsional indices, adjusting for age, body mass index (BMI), blood pressure (SBP and DBP, separately), pulse rate, fasting blood glucose, total cholesterol, HDL, renal function (eGFR) and comorbidities [including hypertension, diabetes, history of coronary artery disease (CAD), active smoking, and regular exercise; Table 2]. In a subset (*n* = 448) of women with onset of menopause timing available, we tested for associations of key deformity indices (torsion, sphericity, GCS, and GLS) with menopause duration as an ordinal variable using linear trend testing for continuous variables (Figure 3). For comparisons between any two categories (e.g., men vs. women, pre- vs. post-menopausal women), we used the unpaired *t*-test for continuous variables. Univariate association between estradiol and key deformity indices were performed using linear regression in a subset of women with available measurements (281/669, 20.0%) (Supplementary Figure 2). Time-to-event analyses were performed using a multivariable Cox proportional hazards model by considering variables with significant univariable *p*-values (*P* < 0.1) and *a priori* choice of important clinical factors to obtain the most parsimonious model, in the absence of violation of the proportion hazard assumption. The primary endpoint was incident heart failure. Secondary endpoint was the composite outcomes of all-cause mortality or incident heart failure was censored at the last date of follow-up, whichever is earlier, in patients who did not have an event (Supplementary Figure 4).

**TABLE 1:** Baseline demographic characteristics of study participants, stratified by sex and menopause status.

	All (*n* = 4,051)			Women (*n* = 1,418)		
Number, *n*	Men (*n* = 2,633)	Women (*n* = 1,418)	*p*-value	Pre-menopausal (*n* = 749)	Post-menopausal (*n* = 669)	*p*-value
**Baseline characteristics**						
Age, years	49.1 ± 10.3	51.3 ± 11.6	< 0.001	42.7 ± 7.3	60.8 ± 7.2	< 0.001
Body weight, kg	72.5 ± 10.9	57.0 ± 9.2	< 0.001	56.5 ± 9.1	57.6 ± 9.2	0.02
BSA, m^2^	1.98 ± 0.17	1.69 ± 0.14	< 0.001	1.69 ± 0.14	1.69 ± 0.15	0.51
BMI, kg/m^2^	25.0 ± 3.3	23.1 ± 3.7	< 0.001	22.3 ± 3.5	24.0 ± 3.7	< 0.001
Systolic blood pressure, mmHg	124.8 ± 15.9	119.9 ± 18.6	< 0.001	113.3 ± 15.0	127.4 ± 19.5	< 0.001
Diastolic blood pressure, mmHg	77.7 ± 10.7	72.0 ± 10.5	< 0.001	69.4 ± 9.9	74.9 ± 10.4	< 0.001
Heart rate, min/s	66.4 ± 11.4	66.2 ± 10.5	0.58	66.8 ± 10.7	65.4 ± 10.3	0.12
Laboratory data						
Fasting glucose, mg/dL	103.4 ± 23.2	97.8 ± 20.7	< 0.001	94.0 ± 16.5	102.3 ± 24.2	< 0.001
Total cholesterol, mg/dL	203.0 ± 35.4	204.7 ± 37.9	0.17	194.1 ± 34.1	217.4 ± 38.3	< 0.001
HDL-c, mg/dL	49.6 ± 12.3	63.6 ± 16.1	< 0.001	64.3 ± 15.4	62.8 ± 16.8	0.08
LDL-c, mg/dL	133.9 ± 32.6	127.6 ± 35.1	< 0.001	117.9 ± 31.2	139.0 ± 36.1	< 0.001
Triglyceride, mg/dL	149.6 ± 103.7	109.8 ± 86.3	< 0.001	97.1 ± 83.1	125.0 ± 87.7	< 0.001
eGFR, mL/min/1.73 m^2^	85.7 ± 14.5	93.8 ± 18.9	< 0.001	98.4 ± 19.0	88.4 ± 17.5	< 0.001
hs-CRP, mL/L (25th∼75th)	0.098 (0.048∼0.22)	0.075 (0.037∼0.19)	0.18	0.059 (0.028∼0.16)	0.094 (0.047∼0.20)	< 0.001
NT-ProBNP, ng/mL (25th∼75th)	20.99 (9.60∼41.1)	44.3 (25∼73.7)	< 0.001	38.03 (20.05∼62.55)	53.0 (31.1∼86.3)	< 0.001
**Sex hormones**						
FSH, mIU/mL (*n* = 281)	—	29.4 ± 25.0	−	14.7 ± 10.6	50.8 ± 24.6	< 0.001
E2, ng/dL (*n* = 281)	—	56.8 ± 71.1	−	87.9 ± 78.3	11.3 ± 4.0	< 0.001
**Lifestyle/Medical history**						
Active smoking, %	297 (11.3%)	40 (2.8%)	< 0.001	25 (3.3%)	15 (2.2%)	0.21
Hypertension, %	474 (18.0%)	239 (16.9%)	0.36	41 (5.5%)	198 (29.6%)	< 0.001
Diabetes, %	163 (6.2%)	103 (7.3%)	0.19	27 (3.6%)	76 (11.4%)	< 0.001
Hyperlipidemia medication, %	218 (8.3%)	150 (10.6%)	0.02	34 (4.5%)	116 (17.3%)	< 0.001
Coronary artery disease, %	98 (3.7%)	56 (4.0%)	0.72	10 (1.3%)	46 (6.9%)	< 0.001

*Data presented as mean ± SD. BMI, body mass index; SBP, systolic blood pressure; DBP, diastolic blood pressure; E2, estradiol; FSH, follicle-stimulating hormone; HDL, high-density lipoprotein; LDL, low-density lipoprotein; eGFR, estimated glomerular filtration rate; hs-CRP, high-sensitivity C- Reactive Protein; NT-ProBNP, N-terminal pro b-type natriuretic peptide; CAD, coronary artery disease.*

**TABLE 2:** Sex differences and menopause relating to cardiac geometry, deformations and multivariable regression model.

	Mean				Multi-variable models			
Number, *n*	Men (*n* = 2633)	Pre-menopausal	Post-menopausal	*p*-value	Menopause status (SBP model) (reference: pre-menopause)		Menopause status (DBP model) (reference: Pre-menopause)	
		Women (*n* = 749)	Women (*n* = 669)	ANOVA	Coef. (95% CI)	*p*-value	Coef. (95% CI)	*p*-value
**Ventricular structure and function**								
IVS, mm	9.2 ± 1.0	8.2 ± 1.0^†^	8.9 ± 1.1^†^*	<0.001	0.28 (0.12, 0.45)	0.001	0.27 (0.10, 0.44)	0.001
LVPW, mm	9.3 ± 0.9	8.2 ± 0.9^†^	8.9 ± 1.0^†^*	<0.001	0.21 (0.06, 0.36)	0.007	0.19 (0.06, 0.36)	0.012
LVM, g	152.6 ± 28.3	113.4 ± 24.8^†^	132.0 ± 29.8^†^*	<0.001	6.2 (2.1, 10.3)	0.003	5.9 (1.8, 10.0)	0.005
LVMi, g/m^2※^	77.0 ± 13.3	66.9 ± 12.5^†^	78.0 ± 16.0*	<0.001	4.46 (2.09, 6.84)	< 0.001	4.2 (1.85, 6.60)	0.001
LV M/V ratio, gm/mL	1.91 ± 0.26	1.72 ± 0.26^†^	1.90 ± 0.30*	<0.001	0.05 (0.01, 0.1)	0.02	0.05 (0.01, 0.1)	0.03
Sphericity	0.60 ± 0.06	0.60 ± 0.07	0.63 ± 0.06^†^*	<0.001	0.02 (0.01, 0.03)	0.001	0.02 (0.01, 0.03)	0.001
LVEDVi, mL/m^2※^	40.5 ± 5.8	39.2 ± 6.4^†^	41.4 ± 7.1^†^*	<0.001	1.28 (0.12, 2.44)	0.031	1.23 (0.07, 2.39)	0.038
LVESVi, mL/m^2※^	15.4 ± 3.0	14.3 ± 3.1^†^	15.1 ± 3.5*	<0.001	0.39(−0.18, 0.96)	0.177	0.38 (−0.19, 0.95)	0.19
SVi, mL/m^2※^	25.0 ± 4.1	24.9 ± 4.5	26.2 ± 4.9^†^*	<0.001	0.89 (0.079, 1.69)	0.032	0.85 (0.04, 1.66)	0.04
LV ejection fraction,%	61.90 ± 5.0	63.6 ± 5.0^†^	63.5 ± 5.2^†^	0.001	0.22 (−0.67, 1.20)	0.63	0.20 (−0.69, 1.09)	0.66
Deceleration time, ms	203.4 ± 38.3	189.1 ± 35.7^†^	217.2 ± 40.7^†^*	<0.001	6.4 (−0.11, 12.92)	0.054	6.7 (0.18, 13.1)	0.044
Mitral e’ (mean), cm/sec	9.28 ± 2.27	10.96 ± 2.20^†^	7.64 ± 1.75^†^*	<0.001	−0.72 (−1.02, −0.43)	<0.001	−0.69 (−0.99, −0.40)	<0.001
LV E/e’ (mean)	7.55 ± 2.35	7.55 ± 2.02	9.77 ± 2.86^†^*	<0.001	0.39 (−0.01, 0.79)	0.053	0.40 (−0.002, 0.81)	0.051
LV E/e’ (mean) > 14,%	53 (2.0%)	7 (0.9%)	50 (7.5%)	<0.001				
**Ventricular deformations**								
GLS,%	19.79 ± 1.72	21.33 ± 1.85^†^	20.34 ± 2.00^†^*	<0.001	−0.37 (−0.70, −0.04)	0.026	−0.37 (−0.70, −0.04)	0.028
GLS < 16,%	32 (1.2%)	3 (0.4%)	8 (1.2%)	0.15				
GCS,%	21.14 ± 3.58	21.62 ± 3.56^†^	21.87 ± 4.09†	<0.001	0.06 (−0.62, 0.74)	0.863	0.08 (−0.61, 0.77)	0.822
Basal rotation,°	−5.4 ± 2.8	−5.2 ± 2.9	−6.3 ± 3.1^†^*	<0.001	−0.46 (−0.99, 0.08)	0.093	−0.48 (−1.02, 0.05)	0.078
Apical rotation,°	9.3 ± 4.5	8.5 ± 4.2^†^	9.6 ± 4.8*	<0.001	0.78 (−0.04, 1.60)	0.062	0.78 (−0.04, 1.62)	0.063
Twist,°	13.8 ± 5.3	12.4 ± 5.1^†^	15.1 ± 5.5^†^*	<0.001	1.47 (0.51, 2.42)	0.003	1.49 (0.53, 2.45)	0.002
Torsion,°/cm	2.14 ± 0.86	2.10 ± 0.88	2.62 ± 1.01^†^*	<0.001	0.36 (0.19, 0.53)	<0.001	0.25 (0.12, 0.39)	<0.001
Torsion-CS,°/cm%	0.102 ± 0.04	0.100 ± 0.04	0.122 ± 0.05^†^*	<0.001	0.018 (0.01, 0.027)	<0.001	0.018 (0.01, 0.027)	<0.001
**Atrial structure, function and deformations**								
LA volume index (BSA), mL/m^2※^	15.5 ± 5.5	15.2 ± 5.0	18.6 ± 6.8^†^*	<0.001	1.03 (0.13, 1.94)	0.025	0.84 (−0.15, 1.84)	0.096
LA volume index (BSA) > 34 mL/m^2^, %	48 (2%)	12 (1.6%)	49 (7.5%)	<0.001				
LA volume index (ht^2.7^), mL/ht^2.7^	7.4 ± 2.9	7.4 ± 2.7	9.7 ± 4.0^†^*	<0.001	0.63 (0.13, 1.13)	0.014	0.47 (−0.052, 0.98)	0.078
LA emptying fraction, %	56.2 ± 12.2	58.3 ± 11.6^†^	56.7 ± 12.1^†^*	<0.001	2.31 (0.38, 4.24)	0.019	2.06 (−0.04, 4.16)	0.054
PALS, %	37.1 ± 8.0	40.9 ± 7.4^†^	34.7 ± 7.9^†^*	<0.001	−2.39 (−3.67, −1.11)	<0.001	−2.53 (−3.83, −1.24)	<0.001
PALS < 23, %	98 (3.7%)	7 (0.9%)	41 (6.1%)	<0.001				
LA Stiffness, %^–1^	0.22 ± 0.10	0.19 ± 0.07^†^	0.30 ± 0.14^†^*	<0.001	0.028 (0.01, 0.05)	0.002	0.066 (0.01, 0.13)	0.034

Adjusted for age, BMI, heart rate, BP (SBP or DBP), fasting blood glucose, total cholesterol, HDL, eGFR, hypertension, diabetes, CAD, and active smoking. ^†^p < 0.05 compared to men, *p < 0.05 compared to pre-menopausal women. *BMI was not included in models.

*BMI, body mass index; SBP, systolic blood pressure; HDL, high-density lipoprotein; eGFR, estimated glomerular filtration rate; CAD, coronary artery disease; LV, left ventricular; IVS, interventricular septum; LVMi, LV mass indexed to body surface area; LV M/V, LV mass-to-volume ratio; LVEDVi, indexed LV end-diastolic volume; LVESVi, indexed LV end-systolic volume; SVi: indexed LV stroke volume; ED, end-diastole; ES, end-systole; GLS, global longitudinal strain; GCS, global circumferential strain; CS, circumferential strain.*

In sensitivity analyses, we performed propensity matched comparisons of: (1) younger men vs. pre-menopausal women, (2) elderly men vs. post-menopausal women, and (3) pre- vs. post-menopausal women (Supplementary Table 1). Propensity scores were derived and 1:1 matching performed separately for each of the 3 group comparisons, accounting for age, BMI, blood pressure, pulse rate, fasting blood glucose, total cholesterol, HDL, eGFR, hypertension, diabetes, CAD, active smoking, and regular exercise. Our propensity score method adopted a macro algorithm (1: N Case-Control Match on Propensity Score) using a SAS macro (SUGI29 Proceedings, 2004) with nearest neighbor caliper width of 0.25. The matching procedure was performed by with SAS Version 9.4 (SAS Institute, Cary, NC).

All statistical analysis was performed using STATA 13 and SPSS 21 software. A 2-sided value of *p* < 0.05 was taken to indicate statistical significance for all analyses.

## Results

### Baseline Characteristics

Among 4051 asymptomatic participants (mean age 49.84 ± 10.81, 35% women; Table 1), women were older, with lower BMI, lower blood pressure, lower fasting blood glucose, better lipid profiles, higher N-terminal pro-B-type natriuretic peptide (NT-proBNP) levels, and better renal function; and were less likely to be active smokers compared to men (Table 1, all *p* < 0.05). Compared to pre-menopausal women, post-menopausal women had higher blood pressure, higher fasting glucose, worse lipid profiles, poorer renal function, and more prevalent hypertension (5.3-fold), diabetes (3.2-fold), hyperlipidemia (3.8-fold), and CAD (5.3-fold) (Table 1, all *p* < 0.05).

### Impact of Sex and Menopause on Cardiac Structure and Mechanics

Women overall exhibited slightly greater LV sphericity (0.61 ± 0.07 vs. 0.60 ± 0.06), smaller LV volumes, marginally higher LV ejection fraction (63.6 vs. 61.9%), higher LV E/e’ (8.6 ± 2.7 vs. 7.5 ± 2.4), better LV GLS (20.87 ± 1.98 vs. 19.79 ± 1.72%), higher LV GCS, and greater LV torsion (2.34 ± 0.98°/cm vs. 2.14 ± 0.86°/cm) than men, along with larger LA volumes, marginally higher PALS and higher LA stiffness (0.25 ± 0.12 vs. 0.22 ± 0.10, all *p* < 0.001) (Table 2). After adjusting for clinical covariates, women were more likely than men to have spherical LV remodeling, worse LV e’, greater LV torsion and higher LA stiffness (Table 2).

Post (vs. pre) -menopausal women demonstrated higher LV sphericity (0.63 ± 0.06 vs. 0.60 ± 0.07), worse diastolic functional indices (lower e’ and higher E/e’), larger LV rotational/torsional mechanics, as well as larger LA volume (indexed to BSA and height) and lower LA deformation (9.7 ± 4.0 vs. 7.4 ± 2.7) (Table 2, all *p* < 0.05). Following multivariable adjustment, the associations of post-menopausal status with greater LV sphericity (0.02, 95%CI 0.01, 0.03), higher indexed LV mass (LVMi), lower mitral e’, lower LV GLS (0.37, 95%CI 0.04–0.70), higher LV torsion, larger LA volume, worse PALS (∼2.4-fold) and greater LA stiffness (0.028, 95%CI 0.01–0.05) remained significant (Table 2 and Supplementary Table 4, all *p* < 0.05). Findings were consistent in sensitivity analyses which excluded individuals with CAD and in propensity-matched comparisons between (1) younger men vs. pre-menopausal women, (2) elderly men vs. post-menopausal women and (3) pre- vs. post-menopausal women (Supplementary Figure 1 and Supplementary Table 1).

### Association of Menopause Duration and Estradiol Level With Cardiac Structure and Mechanics

Among 448 (70%) women in whom duration of menopause was available, increasing duration of menopause (per 5-year increment) was independently associated with greater LVMi, greater LV sphericity (0.05, 95%CI: 0.02, 0.09), lower mitral e’, higher mitral E/e’, worse LV GLS (1.31, 95%CI: 0.61, 2.02), larger indexed LA volume, lower PALS (−4.43, 95%CI: −7.17, −1.70), greater LA stiffness and augmented LV torsion (1.02, 95%CI: 0.64, 1.39, all *p* < 0.05) (Figure 3).

In a subset of 281 women (19.8%), irrespective of menopause status, estradiol measurements were available (mean age: 47.7 ± 9.0 years). Lower estradiol level correlated with more impaired LV diastolic function (lower mitral e’ and higher E/e’) and lower LV GLS (*r* = 0.21) and worse PALS (*r* = 0.39 for GLS and PALS, respectively) but greater LVMi, (*r* = −0.20), LV sphericity (*r* = −0.23), LV torsion (*r* = −0.21) and LA stiffness (*r* = −0.27) (Supplementary Figure 2, all *p* < 0.05), even after accounting for age and clinical covariates (Supplementary Table 2) and excluding patients with CAD.

In a separate cohort of 320 women (mean age 49.0 ± 10.4 years) recruited in the outpatient clinic during routine attendance for health screening for cardiac conditions, combined estradiol blood sampling and comprehensive echocardiography were available in 320 subjects (mean age: 49.0 ± 10.4) free from HF symptoms. Lower estradiol levels were associated with greater LV sphericity, worsened mitral e’, lower GLS, worse PALS and higher torsion, similar to the main cohort (Supplementary Figure 3).

We further explored the association between cardiac structural and functional changes with menopause status and estradiol levels (Supplementary Table 3). The structural parameters of increased LVMi and increased LV sphericity were independently associated with functional parameters of lower LV e’, higher mitral E/e’ and worse PALS (all *p* < 0.05). Increased LVMi was further independently related to GLS decline (Supplementary Table 3).

### Associations of Cardiac Mechanics and Outcomes Among Post-menopausal Women

In the entire cohort, there were 102 primary events of HF incidence and 113 composite endpoints of all-cause death and incident HF over a median follow up period of 2.9 years (IQR: 2.2∼3.9 years). Of which, 37 (5.5%) HF incidence and 34 (5.1%) composite events occurred among 669 post-menopausal women. Higher LA stiffness, lower PALS and reduced GLS predicted the risk of the primary outcome of HF incidence as well as the secondary outcome of composite events (Table 3). When adjusted for clinical covariates (i.e., all conventional cardiovascular confounders, LVEF, E/e’, and LAEF separately), greater LV sphericity [adjusted hazard ratio (aHR) 1.04, 95%CI 1.00-1.07], impaired GLS (aHR 0.87, 95%CI 0.78-0.97), reduced PALS (aHR 0.94, 95%CI 0.90-0.99) and higher LA stiffness (aHR 10.5, 95%CI 1.69-64.6) were independently associated with the primary outcome of HF hospitalizations in post-menopause (all *p* < 0.05). Results were consistent with composite outcomes of HF hospitalizations and 1-year all-cause mortality (Table 3 and Supplementary Figure 4) and when variables were analyzed dichotomously using clinical abnormality cut-offs (Table 4).

**TABLE 3:** Prognostic values of deformational indices by multivariable Cox regression model in post-menopausal women (*n* = 669).

		Univariate		Multi-variate									
				Model 1		Model 2		Model 3		Model 4		Model 5	
		HR (95% CI)	*p-*value	HR (95% CI)	*p*-value	HR (95% CI)	*p*-value	HR (95% CI)	*p*-value	HR (95% CI)	*p*-value	HR (95% CI)	*p*-value
**Types of events: HF incidence**													
LA deformation	PALS,%	0.90 (0.85–0.95)	<0.001	0.92 (0.87–0.97)	<0.001	0.93 (0.89–0.97)	0.01	0.93 (0.89–0.98)	0.02	0.94 (0.90–0.99)	0.03	0.94 (0.90–0.99)	0.03
	LA Stiffness,%^–1^	23.4 (6.2–88.6)	<0.001	18.7 (3.82–86.4)	<0.001	12.2 (2.36–63.0)	0.02	11.7 (1.80–76.4)	0.01	—	—	10.5 (1.69–64.6)	0.012
LV Deformation/Geometry	Sphericity	1.59 (1.22–2.09)	0.001	1.46 (1.10–1.93)	0.008	1.34 (1.08–1.67)	0.01	1.33 (1.02–1.75)	0.02	1.32 (1.00–1.77)	0.03	1.45 (1.06–1.96)	0.019
	GLS,%	0.78 (0.72–0.86)	<0.001	0.85 (0.77–0.94)	0.001	0.86 (0.77–0.97)	0.012	0.86 (0.77–0.97)	0.012	0.87 (0.77–0.97)	0.015	0.87 (0.78–0.97)	0.015
	GCS,%	0.91 (0.83–0.99)	0.038	0.93 (0.84–0.99)	0.045	0.97 (0.88–1.06)	0.49	0.97 (0.88–1.06)	0.55	0.97 (0.88–1.06)	0.47	0.96 (0.88–1.06)	0.44
	Torsional Mechanics, °/cm	1.03 (0.72–1.46)	0.87	1.03 (0.74–1.47)	0.93	1.06 (0.73–1.54)	0.75	1.07 (0.73–1.56)	0.73	1.06 (0.73–1.54)	0.76	1.08 (0.75–1.58)	0.67
**Types of events: HF incidence + Any Death**													
LA deformation	PALS,%	0.91 (0.87–0.96)	<0.001	0.93 (0.88–0.97)	0.001	0.94 (0.90–0.99)	0.02	0.94 (0.90–0.99)	0.023	0.94 (0.90–0.99)	0.026	0.94 (0.89–0.99)	0.018
	LA Stiffness,%^–1^	26.2 (7.1–97.1)	<0.001	17.7 (3.74–83.2)	<0.001	12.4 (1.79–85.8)	0.011	11.8 (1.67–82.6)	0.013	—	—	13.2 (1.99–87.6)	0.008
LV deformation/geometry	Sphericity	1.05 (1.01–1.08)	0.004	1.04 (1.00–1.07)	0.019	1.04 (1.00–1.07)	0.03	1.03 (1.00–1.07)	0.032	1.03 (1.00–1.07)	0.043	1.04 (1.01–1.07)	0.024
	GLS,%	0.79 (0.71–0.87)	<0.001	0.83 (0.75–0.93)	0.001	0.84 (0.74–0.96)	0.009	0.84 (0.74–0.96)	0.009	0.85 (0.75–0.97)	0.015	0.85 (0.75–0.96)	0.01
	GCS,%	0.90 (0.82–0.98)	0.018	0.90 (0.83–0.99)	0.025	0.93 (0.82–1.04)	0.21	0.97 (0.88–1.07)	0.55	0.96 (0.88–1.06)	0.46	0.97 (0.88–1.06)	0.48
	Torsional Mechanics, °/cm	1.16 (0.81–1.67)	0.42	1.13 (0.79–1.61)	0.51	1.21 (0.82–1.80)	0.34	1.18 (0.79–1.76)	0.42	1.18 (0.79–1.77)	0.41	1.21 (0.81–1.81)	0.34

*Model 1: adjusted for age. Model 2: adjusted for age, BMI, heart rate, SBP, eGFR, hypertension, diabetes, CAD and active smoking. Model 3: adjusted for age, BMI, heart rate, SBP, eGFR, hypertension, diabetes, CAD and active smoking, LVEF. Model 4: adjusted for age, BMI, heart rate, SBP, eGFR, hypertension, diabetes, CAD and active smoking, E/e’. Model 5: adjusted for age, BMI, heart rate, SBP, eGFR, hypertension, diabetes, CAD and active smoking, LAEF.*

**TABLE 4:** Prognostic values of deformational indices (using cutoffs) by multivariable Cox regression model in post-menopausal women (*n* = 669).

Types of events: HF incidence	Univariate		Multi-variate									
			Model 1		Model 2		Model 3		Model 4		Model 5	
	HR (95% CI)	*p-*value	HR (95% CI)	*p*-value	HR (95% CI)	*p*-value	HR (95% CI)	*p*-value	HR (95% CI)	*p*-value	HR (95% CI)	*p*-value
PALS < 23, %	2.98 (1.34–7.55)	<0.001	2.76 (1.18–7.49)	0.003	1.37 (1.02–3.57)	0.02	1.38 (1.02–3.64)	0.01	1.31 (1.02–3.47)	0.03	1.32 (1.01–3.49)	0.03
GLS < 16, %	12.3 (4.32–34.80)	<0.001	5.90 (1.94-17.96)	0.002	6.43 (2.03–20.4)	0.002	6.47 (2.04–20.5)	0.002	6.14 (1.80–20.9)	0.004	5.80 (1.79–18.8)	0.003

*Model 1: adjusted for age. Model 2: adjusted for age, BMI, heart rate, SBP, eGFR, hypertension, diabetes, CAD and active smoking. Model 3: adjusted for age, BMI, heart rate, SBP, eGFR, hypertension, diabetes, CAD and active smoking, LVEF. Model 4: adjusted for age, BMI, heart rate, SBP, eGFR, hypertension, diabetes, CAD and active smoking, E/e’. Model 5: adjusted for age, BMI, heart rate, SBP, eGFR, hypertension, diabetes, CAD and active smoking, LAEF.*

## Discussion

In this study using speckle-based tracking to comprehensively evaluate atrioventricular mechanics in a large asymptomatic population, women, compared to men, had better LV systolic function, greater LV sphericity and torsion, as well as better LA mechanics but poorer LV diastolic function. Female menopause and decreasing estradiol levels were associated with greater LV/LA remodeling coupled with declines in LV longitudinal and LA deformation. These structural and functional changes were accentuated with increasing years of menopause, independent of age and cardiovascular risk factors. Furthermore, among post-menopausal women, declining atrioventricular mechanics were independently associated with clinical outcomes. Collectively, these data contribute to our understanding of sex differences in HF and the predominance of postmenopausal women among patients with HFpEF.

Our findings of smaller LV dimensions, greater LV sphericity and concentricity, larger LA size and worse LV diastolic function despite better LV systolic function in women compared to men are consistent with previous studies (16–18). Our results extend prior data by showing that independent of age and cardiovascular risk factors, female menopause is associated with greater LV and LA structural remodeling in tandem with subtle declines in LV and LA longitudinal deformation, despite preserved LV EF. Of note, while LV longitudinal function declined, LV torsional function increased in post-menopausal women. Torsion has shown to preserve global LV pump function (LVEF) (24, 25) by mechanically increasing the wringing motion of the heart and mechanistically favoring more efficient O_2_ and energy utilization (26, 27). The synergistic effects of a smaller LV size (dimensions and volumes), shorter LV long axis length and potentially greater LV sphericity in women could contribute to enhanced LV torsion by having more horizontally oriented oblique myocardial fibers between the subendocardium and subepicardium. This, (1) generates a greater net angle difference, which favors increased torsion (lever-arm theory) and (2) enhances shearing of subendocardial fibers toward the LV cavity during contraction, which promotes LV wall thickening. Our study further adds that post-menopausal women exhibit lower longitudinal atrial mechanics (15% PALS reduction) and increased atrial stiffness (40% increase), consistent with the known predisposition of elderly women to LV diastolic dysfunction despite preserved LVEF and atrial fibrillation (30).

**FIGURE 4:**
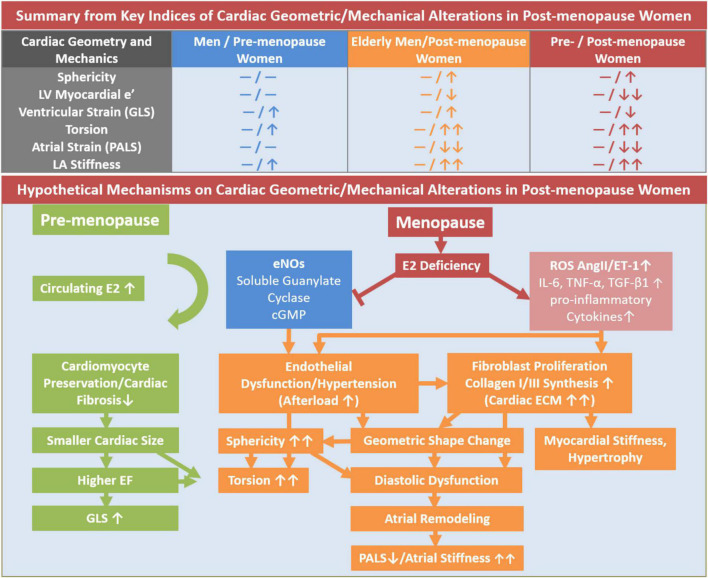
Illustrative summary of hypothetical mechanisms of LV geometric and mechanical alterations in post-menopausal women in contrast to men and pre-menopausal women. cGMP, cyclic GMP; eNOS, endothelial nitric oxide synthase; GLS, global longitudinal strain; IL-6, cytokine interleukin-6; PALS, peak atrial longitudinal strain; TGF-β, transforming growth factor; TNF, tumor necrosis factor.

To our knowledge this is the first description of the “dose response” association of increasing duration of menopause and decreasing circulating estradiol levels with accentuated cardiac structural and functional changes among women. Notably, we found that LV/LA deformational changes began immediately after menopause onset (<5 years post-menopause) with continued progressive deterioration, correlating with lower estradiol levels (Supplementary Table 2) and independent of age and clinical covariates. Early onset of menopause has been linked to increased risk of HF. Studies have attributed the reduced lifetime exposure to estrogen and shorter reproductive duration to greater HF risk in women (12, 13, 31, 32). Chinese American women in early menopause are at higher risk of cardiac remodeling compared to other ethnicities (33). Menopause and shorter reproductive duration from early onset of menopause have been proposed to be a risk factor for HFpEF in women (9–12, 14, 23). There is biological plausibility for the role of declining estrogen levels in mediating the cardiac structural and functional changes in post-menopausal women. Estrogen reduces myocardial cell loss by inhibiting apoptosis, minimizes reactive oxygen species related cardiac injury (28), reduces Ca^2+^ transient through negative inotropic effects (29), decreases glucose utilization (through upregulation of nitric oxide synthases) and instead favors fatty acid oxidation for higher energy yield (Figure 4) (34). Atrial tissue is also more susceptible to oxidative stress, inflammation and pathological fibrotic replacement (i.e., TGF-β/Smad3 signaling) in estrogen-deficient conditions (Figure 4) (25, 35, 36).

Importantly, PALS and LV GLS are highly sensitive and relatively load-independent surrogate measures of LA and LV function, respectively. Our data suggest that deformation measures of PALS, LA stiffness index and LV GLS, with respective cut-offs of 31.6, 0.32, and 18.2%, may be more sensitive in detecting subclinical changes in kinetics, even when conventional LA (i.e., LA emptying fraction) and LV markers (i.e., LV ejection fraction or E/e’) remain unchanged.

## Study Limitation

The cross-sectional nature of this study precludes conclusions regarding causality. Menopause status and duration were determined via questionnaire, which was a feasible method for a large screening population. The explorative findings on estradiol levels and cardiac deformation indices impedes generalizable inferences and should be further validated in future prospective studies. Finally, our findings from an exclusively Asian population may not be generalizable to other ethnicities and may need to be validated from future studies, although aging and menopause are universal occurrences, and our data are consistent with that from other populations.

## Conclusion

Women have better LV systolic function, greater LV sphericity and torsion, as well as better LA mechanics but poorer LV diastolic function compared to men. Female menopause is associated with greater LV/LA remodeling coupled with declines in LV longitudinal and LA function independent of age and cardiovascular risk factors. The accentuation of these structural and function changes with increasing years of menopause and lower levels of estradiol, along with their independent prognostic value in post-menopausal women, may contribute to our understanding of sex differences in heart failure.

## Data Availability Statement

The datasets presented in this article are not readily available because Data analyzed for this study are available from the MacKay Memorial Hospital Institutional Data Access/Ethics Committee for investigators who are bound by confidentiality agreements. Contact details: Mackay Memorial Hospital Institutional Review Board Address: No. 92, Sec. 2, Zhongshan N. Rd., Taipei City 10449, Taiwan Tel:02-25433535#3486∼3488, mmhirb82@gmail.com. Requests to access the datasets should be directed to mmhirb82@gmail.com.

## Ethics Statement

The studies involving human participants were reviewed and approved by the Mackay Memorial Hospital (IRB No: 18MMHIS109). Informed consent to participate in this study was waived from Institution Review Board due to retrospective study design.

## Author Contributions

C-LH and CL: conceptualization and validation. K-TS and C-LH: data curation and formal analysis. C-LH: funding acquisition and project administration. C-LH, CC, and BB: investigation. K-TS, CC, C-IL, J-PT, Y-HL, C-CH, S-YT, C-HY, T-CH, and C-HS: data collection and methodology. C-LH, H-IY, and C-LH: supervision. CC: visualization. K-TS and CC: writing—original draft. CC, C-LH, and CL: manuscript editing. All authors contributed to the article and approved the submitted version.

## Conflict of Interest

The authors declare that the research was conducted in the absence of any commercial or financial relationships that could be construed as a potential conflict of interest.

## Publisher’s Note

All claims expressed in this article are solely those of the authors and do not necessarily represent those of their affiliated organizations, or those of the publisher, the editors and the reviewers. Any product that may be evaluated in this article, or claim that may be made by its manufacturer, is not guaranteed or endorsed by the publisher.
