# Supplementary material for: Association of Female Menopause With Atrioventricular Mechanics and Outcomes
Source: Front Cardiovasc Med. 2022 Apr 21;9:804336. doi: 10.3389/fcvm.2022.804336 (PMC9068967; doi:10.3389/fcvm.2022.804336)

**Supplemental Materials**

**(Tables)**

**Supp Table 1. Baseline demographic characteristics of study participants, stratified by sex and menopause status**

|  | **All (n=4051)** | |  | **Women (n=1418)** | |  |
| --- | --- | --- | --- | --- | --- | --- |
| **Number, n** | **Men (n= 2633)** | **Women (n= 1418)** | ***p value*** | **Pre-menopausal (n= 749)** | **Post-menopausal (n= 669)** | ***p value*** |
| ***Baseline Characteristics*** |  |  |  |  |  |  |
| Age, years | 49.1±10.3 | 51.3±11.6 | <0.001 | 42.7±7.3 | 60.8±7.2 | <0.001 |
| Body weight, kg | 72.5±10.9 | 57.0±9.2 | <0.001 | 56.5±9.1 | 57.6±9.2 | 0.02 |
| BSA, m^2^ | 1.98±0.17 | 1.69±0.14 | <0.001 | 1.69±0.14 | 1.69±0.15 | 0.51 |
| BMI, kg/m^2^ | 25.0±3.3 | 23.1±3.7 | <0.001 | 22.3±3.5 | 24.0±3.7 | <0.001 |
| Systolic blood pressure, mmHg | 124.8±15.9 | 119.9±18.6 | <0.001 | 113.3±15.0 | 127.4±19.5 | <0.001 |
| Diastolic blood pressure, mmHg | 77.7±10.7 | 72.0±10.5 | <0.001 | 69.4±9.9 | 74.9±10.4 | <0.001 |
| Heart rate, min/sec | 66.4±11.4 | 66.2±10.5 | 0.58 | 66.8±10.7 | 65.4±10.3 | 0.12 |
| ***Laboratory Data*** |  |  |  |  |  |  |
| Fasting glucose, mg/dL | 103.4±23.2 | 97.8±20.7 | <0.001 | 94.0±16.5 | 102.3±24.2 | <0.001 |
| Total cholesterol, mg/dL | 203.0±35.4 | 204.7±37.9 | 0.17 | 194.1±34.1 | 217.4±38.3 | <0.001 |
| HDL-c, mg/dL | 49.6±12.3 | 63.6±16.1 | <0.001 | 64.3±15.4 | 62.8±16.8 | 0.08 |
| LDL-c, mg/dL | 133.9±32.6 | 127.6±35.1 | <0.001 | 117.9±31.2 | 139.0±36.1 | <0.001 |
| Triglyceride, mg/dL | 149.6±103.7 | 109.8±86.3 | <0.001 | 97.1±83.1 | 125.0±87.7 | <0.001 |
| eGFR, mL/min/1.73m^2^ | 85.7±14.5 | 93.8±18.9 | <0.001 | 98.4±19.0 | 88.4±17.5 | <0.001 |
| hs-CRP, ml/L (25^th^ ~ 75^th^) | 0.098 (0.048 ~ 0.22) | 0.075 (0.037 ~ 0.19) | 0.18 | 0.059 (0.028 ~ 0.16) | 0.094 (0.047 ~ 0.20) | <0.001 |
| NT-ProBNP, ng/mL (25^th^ ~ 75^th^) | 20.99 (9.60 ~ 41.1) | 44.3 (25 ~ 73.7) | <0.001 | 38.03 (20.05 ~ 62.55) | 53.0 (31.1 ~ 86.3) | <0.001 |
| ***Sex Hormones*** |  |  |  |  |  |  |
| FSH, mIU/mL (n=281) | — | 29.4±25.0 | — | 14.7±10.6 | 50.8±24.6 | <0.001 |
| E2, ng/dL (n=281) | — | 56.8±71.1 | — | 87.9±78.3 | 11.3±4.0 | <0.001 |
| ***Lifestyle/Medical History*** |  |  |  |  |  |  |
| Active smoking, % | 297 (11.3 %) | 40 (2.8 %) | <0.001 | 25 (3.3 %) | 15 (2.2 %) | 0.21 |
| Hypertension, % | 474 (18.0 %) | 239 (16.9 %) | 0.36 | 41 (5.5 %) | 198 (29.6 %) | <0.001 |
| Diabetes, % | 163 (6.2 %) | 103 (7.3%) | 0.19 | 27 (3.6 %) | 76 (11.4 %) | <0.001 |
| Hyperlipidemia medication, % | 218 (8.3%) | 150 (10.6%) | 0.02 | 34 (4.5%) | 116 (17.3%) | <0.001 |
| Coronary artery disease, % | 98 (3.7%) | 56 (4.0%) | 0.72 | 10 (1.3%) | 46 (6.9%) | <0.001 |

Data presented as mean ± SD. Abbreviations: BMI: body mass index, SBP: systolic blood pressure, DBP: diastolic blood pressure, E2: estradiol, FSH: follicle-stimulating hormone, HDL: high-density lipoprotein, LDL: low-density lipoprotein, eGFR: estimated glomerular filtration rate, hs-CRP: high-sensitivity C- Reactive Protein, NT-ProBNP: N-terminal pro b-type natriuretic peptide, CAD: coronary artery disease,

**Supp.** **Table 2. Demographics and** **echocardiographic measurement and cardiac mechanics based on men, pre-menopausal, and post-menopausal women after propensity matching procedure**

|  | **Men vs. Women** | | | | |  | **Women (Pre- vs. Post-menopause)** | |
| --- | --- | --- | --- | --- | --- | --- | --- | --- |
|  | **Men** | **Pre-menopausal Women** |  | **Elderly Men** | **Post-menopausal Women** |  | **Pre-menopausal Women** | **Post-menopausal Women** |
| **Number, n** | **(n = 462)** | **(n = 462)** |  | **(n = 437)** | **(n = 437)** |  | **(n = 86)** | **(n = 86)** |
| Age, years | 44.2±10.0 | 44.6±6.3 |  | 60.0±8.6 | 59.2±6.2 |  | 51.8±2.1 | 51.7±2.2 |
| BMI, kg/m^2^ | 23.2±2.9 | 23.0±3.5 |  | 24.2±3.1 | 24.2±3.7 |  | 22.8±3.3 | 23.1±3.3 |
| SBP, mmHg | 116.4±13.0 | 116.2±15.3 |  | 127.3±16.5 | 125.9±19.4 |  | 120.3±15.7 | 119.2±17.4 |
| Heart Rate, min/sec | 74.0±11.4 | 73.8±11.0 |  | 73.4±11.4 | 73.2±11.4 |  | 73.5±9.3 | 74.2±9.5 |
| Nt-ProBNP, ng/mL (25^th^ ~ 75^th^)† | 15.7 (6.6 ~ 32.1) | 39.6 (22.7 ~ 62.9)^*^ |  | 28.7 (14.1 ~ 28.7) | 50.8 (30.9 ~ 83.3) ^*^ |  | 38.4 (20.7 ~ 66.4) | 46.5 (28.2 ~ 73.9) |
| **Conventional Echocardiography** |  |  |  |  |  |  |  |  |
| LA Volume index (m^2^), ml/m^2^ | 13.8±4.9 | 15.4±4.9^*^ |  | 16.5±5.9 | 18.4±6.7^*^ |  | 14.6±4.5 | 16.8±6.1^*^ |
| LA volume index (ht^2.7^), ml/ht^2.7^ | 6.3±2.5 | 7.6±2.7^*^ |  | 7.9±3.1 | 9.6±3.9^*^ |  | 7.3±2.6 | 8.5±3.3^*^ |
| LA emptying fraction, % | 56.9±12.1 | 57.9±11.4 |  | 56.3±12.4 | 57.3±12.0 |  | 55.7±12.2 | 57.2±11.2 |
| IVS, mm | 8.9±1.0 | 8.3±0.94^*^ |  | 9.3±1.00 | 9.0±1.1^*^ |  | 8.3±1.0 | 8.7±0.9^*^ |
| LVM, g | 143.3±27.4 | 117.4±24.9^*^ |  | 153.3±28.7 | 114.3±24.6^*^ |  | 114.3±24.6 | 125.7±25.6^*^ |
| LVMi, g/m^2^ | 74.4±13.2 | 67.9±12.5^*^ |  | 80.4±14.2 | 78.3±15.0^*^ |  | 67.9±13.2 | 75.2±13.7^*^ |
| LV M/V ratio, g/ml | 1.85±0.26 | 1.75±0.25^*^ |  | 1.94±0.26 | 1.90±0.28^*^ |  | 1.78±0.24 | 1.86±0.23^*^ |
| LVEDVi, ml | 40.5±6.2 | 39.1±6.3^*^ |  | 41.8±6.4 | 41.4±6.9 |  | 38.7±7.8 | 41.2±6.6^*^ |
| LVESVi, ml | 15.6±3.1 | 14.3±3.1^*^ |  | 15.7±3.2 | 15.0±3.3^*^ |  | 14.2±3.3 | 15.3±3.3^*^ |
| LV SVi, ml | 24.9±4.3 | 24.8±4.4 |  | 26.1±4.4 | 26.4±4.8 |  | 24.4±5.4 | 25.9±4.6^*^ |
| LV ejection fraction, % | 61.5±5.0 | 63.5±4.9^*^ |  | 62.4±5.0 | 63.8±4.9^*^ |  | 63.1±4.8 | 63.0±5.3^*^ |
| Deceleration time, ms | 195.3±36.0 | 192.1±36.7 |  | 215.3±43.6 | 221.2±38.4^*^ |  | 200.3±41.3 | 209.1±33.9 |
| Iso-volumic relaxation time, ms | 88.3±13.8 | 85.0±11.7^*^ |  | 94.5±18.4 | 95.7±14.2 |  | 89.3±12.8 | 90.6±10.5 |
| Mitral e’ (mean), cm/sec | 10.42±2.41 | 10.54±2.10 |  | 8.10±1.97 | 7.76±1.78^*^ |  | 9.28±1.71 | 8.75±1.68^*^ |
| LV E/e’ (mean) | 6.75±1.75 | 7.74±2.06^*^ |  | 8.42±2.92 | 9.75±2.83^*^ |  | 8.52±2.26 | 9.07±2.59^*^ |
| Sphericity | 0.60±0.06 | 0.60±0.06 |  | 0.61±0.07 | 0.63±0.06^*^ |  | 0.59±0.06 | 0.63±0.05^*^ |
| **Deformation and Torsion Indices** |  |  |  |  |  |  |  |  |
| GLS,% | -20.20±1.84 | -21.12±1.80^*^ |  | -19.72±1.95 | -20.37±1.92^*^ |  | -21.29±1.72 | -20.63±1.86^*^ |
| GCS,% | -20.92±3.44 | -21.53±3.55^*^ |  | -21.13±3.75 | -21.89±3.97^*^ |  | -21.44±3.80 | -22.07±4.23 |
| Twist, ° | 12.34±5.07 | 12.40±5.11 |  | 14.03±5.42 | 15.00±5.38^*^ |  | 12.79±5.41 | 16.36±5.40^*^ |
| Torsion, °/cm | 1.95±0.82 | 2.08±0.87^*^ |  | 2.19±0.87 | 2.59±0.97^*^ |  | 2.10±0.88 | 2.88±0.99^*^ |
| Torsion-CS, °/cm% | -0.093±0.038 | -0.098±0.039^*^ |  | -0.105±0.04 | -0.121±0.05^*^ |  | -0.10±0.042 | -0.14±0.051^*^ |
| PALS, % | 39.8±8.1 | 39.9±7.3 |  | 36.2±8.5 | 34.9±7.7^*^ |  | 38.9±6.7 | 36.1±6.8^*^ |
| LA Stiffness | 0.18±0.07 | 0.20±0.08^*^ |  | 0.25±0.13 | 0.30±0.14^*^ |  | 0.23±0.09 | 0.26±0.10^*^ |

Data presented as mean ± SD, except Nt-ProBNP (as median and IQR: 25^th^~75th)†; * denotes p <0.05 among respective PSM-matched groups.

Abbreviations: BMI: body mass index, SBP: systolic blood pressure, DBP: diastolic blood pressure, NT-ProBNP: N-terminal pro b-type natriuretic peptide, LV: left ventricular; IVS: interventricular septum, LVPW: left ventricular posterior wall thickness, LVM: LV mass; LVMi: LV mass indexed to body surface area, LV M/V: LV mass-to-volume ratio, LVEDVi: indexed LV end-diastolic volume, LVESVi: indexed LV end-systolic volume, SVi: indexed stroke volume, DT: deceleration time; IVRT: isovolumetric relaxation time, ED: end-diastole, ES: end-systole, e’: early diastolic mitral annular velocity, E/e’: ratio of mitral peak velocity of early filling to early diastolic mitral annular velocity, GLS: global longitudinal strain; GCS: global circumferential strain, Torsion-CS: Torsion indexed to circumferential strain.

**Supp. Table 3. Associations of circulating estradiol (+10 ng/dL) level with measures of cardiac structure and function among 281 women**

| **Menopause Duration Categories** | **Pearson Correlation** | **Coefficient value (unadjusted)** | ***p* value** | **Coefficient value**  **(age- adjusted)** | **Coefficient value**  **(CV adjusted)**^¥^ | ***p* value**^¥^ |
| --- | --- | --- | --- | --- | --- | --- |
|  | **R value** | **Coef. (95% CI)** |  | **Coef. (95% CI)** |  |  |
| **Ventricular Structure/ Function** |  |  |  |  |  |  |
| IVS, mm | -0.17 | -0.03 (-0.04, -0.008) | 0.004 | -0.02 (-0.04, -0.003) | -0.02 (-0.03, 0.003) | 0.11 |
| LVPW, mm | -0.18 | -0.03 (-0.05, -0.01) | 0.003 | -0.03 (-0.05, -0.005) | -0.02 (-0.05, -0.001) | 0.041 |
| LVM, gm | -0.27 | -1.11 (-1.59, -0.64) | <0.001 | -0.93 (-1.41, -0.45) | -0.97 (-1.48, -0.47) | <0.001 |
| LVMi, gm/m^2※^ | -0.27 | -0.67 (-0.95, -0.38) | <0.001 | -0.56 (-0.84, -0.27) | -0.59 (-0.89, -0.29) | <0.001 |
| LV M/V ratio, gm/ml | -0.19 | -0.01 (-0.01, -0.002) | 0.012 | -0.006 (-0.01, -0.0003) | -0.01 (-0.02, -0.001) | 0.034 |
| Sphericity | -0.32 | -0.003 (-0.004, -0.002) | <0.001 | -0.002 (-0.003, -0.001) | -0.002 (-0.003, -0.001) | <0.001 |
| LVEDVi, ml^※^ | -0.20 | -0.30 (-0.48, -0.13) | 0.001 | -0.25 (-0.43, -0.07) | -0.29 (-0.47, -0.12) | 0.001 |
| LVESVi, ml^※^ | -0.17 | -0.11 (-0.19, -0.04) | 0.004 | -0.09 (-0.17, -0.01) | -0.10 (-0.18, -0.02) | 0.018 |
| LV SVi, ml/m^2※^ | -0.18 | -0.19 (-0.32, -0.06) | 0.003 | -0.16 (-0.28, -0.03) | -0.20 (-0.32, -0.08) | 0.78 |
| LV ejection fraction, % | 0.07 | 0.05 (-0.04, 0.14) | 0.26 | 0.06 (-0.03, 0.15) | 0.07 (-0.02, 0.17) | 0.14 |
| Deceleration time, ms | -0.06 | -0.42 (-1.21, 0.38) | 0.31 | 0.03 (-0.77, 0.82) | 0.11 (-0.81, 1.03) | 0.82 |
| Iso-volumic relaxation time, ms | -0.17 | -0.35 (-0.59, -0.10) | 0.005 | -0.17 (-0.41, 0.06) | -0.10 (-0.36, 0.17) | 0.46 |
| Mitral e’ (mean), cm/sec | 0.31 | 0.10 (0.06, 0.13) | <0.001 | 0.07 (0.03, 0.10) | 0.05 (0.02, 0.09) | 0.002 |
| LV E/e’ (mean) | -0.20 | -0.08 (-0.12, -0.03) | 0.001 | -0.05 (-0.09, -0.005) | -0.04 (-0.09, 0.01) | 0.10 |
| GLS, % | 0.21 | 0.05 (0.02, 0.08) | <0.001 | 0.05 (0.02, 0.08) | 0.04 (0.01, 0.08) | 0.007 |
| GCS, % | 0.04 | 0.02 (-0.04, 0.08) | 0.50 | 0.02 (-0.04, 0.08) | 0.03 (-0.04, 0.10) | 0.39 |
| Twist, ° | -0.21 | -0.14 (-0.22, -0.06) | <0.001 | -0.11 (-0.19, -0.03) | -0.10 (-0.19, -0.008) | 0.033 |
| Torsion, °/cm | -0.21 | -0.03 (-0.04, -0.01) | <0.001 | -0.02 (-0.04, -0.006) | -0.02 (-0.04, -0.001) | 0.039 |
| **Atrial Structure/Function** |  |  |  |  |  |  |
| LA Volume index (BSA), ml/m^2※^ | -0.21 | -0.22 (-0.34, -0.09) | <0.001 | -0.20 (-0.32, -0.07) | -0.21 (-0.33, -0.08) | 0.001 |
| LA emptying fraction, % | 0.10 | 0.16 (-0.08, 0.40) | 0.19 | -0.07 (-0.17, 0.32) | -0.001 (-0.33, 0.32) | 0.98 |
| PALS, % | 0.39 | 0.43 (0.31, 0.55) | <0.001 | 0.35 (0.23, 0.47) | 0.27 (0.14, 0.39) | <0.001 |
| LA Stiffness | -0.27 | -0.004 (-0.006, -0.003) | <0.001 | -0.003 (-0.005, -0.001) | -0.003 (-0.005, -0.001) | 0.014 |

**CV adjusted**^¥^: age, BMI, heart rate, and medical histories of hypertension, diabetes, coronary artery disease and renal profile in terms of eGFR.

**Supp. Table 4. Associations of various LV remodeling patterns with key cardiac diastolic and deformational indices in women by multi-variate analysis (n=1418)**

|  | **LVMi**^※^ | | **LVEDVi**^※^ | | **LV M/V** | | **Sphericity** | |
| --- | --- | --- | --- | --- | --- | --- | --- | --- |
|  | **Coef. (95% CI)** | ***p* value** | **Coef. (95% CI)** | ***p* value** | **Coef. (95% CI)** | ***p* value** | **Coef. (95% CI)** | ***p* value** |
| **Mitral e’ (mean), cm/sec** | -0.02 (-0.03, -0.01) | <0.001 | -0.01 (-0.02, 0.008) | 0.42 | -0.90 (-1.20, -0.61) | <0.001 | -3.96 (-5.38, -2.88) | <0.001 |
| **LV E/e’ (mean)** | 0.015 (0.005, 0.02) | 0.002 | 0.015 (-0.004, 0.03) | 0.13 | 0.55 (0.15, 0.95) | 0.007 | 4.02 (2.06, 5.98) | <0.001 |
| **GCS, %** | -0.01 (-0.02, 0.008) | 0.35 | -0.012 (-0.04, 0.02) | 0.46 | 0.02 (-0.66, 0.70) | 0.95 | -1.70 (-5.00, 1.61) | 0.32 |
| **GLS, %** | -0.02 (-0.03, -0.01) | <0.001 | -0.01 (-0.03, 0.005) | 0.20 | -0.61 (-0.99, -0.23) | 0.002 | 0.38 (-1.22, 1.97) | 0.64 |
| **Torsion, °/cm** | -0.01 (-0.01, -0.001) | <0.001 | -0.01 (-0.02, -0.005) | 0.001 | 0.06 (-0.11, 0.23) | 0.49 | 0.92 (0.10, 1.74) | <0.001 |
| **PALS, %** | -0.07 (-0.10, -0.04) | <0.001 | -0.03 (-0.1, 0.03) | 0.27 | -2.45 (-3.74, -1.17) | <0.001 | -7.11 (-13.2, -1.07) | 0.02 |

Adjusted for age, BMI, heart rate, SBP, fasting blood glucose, total cholesterol, HDL, eGFR, hypertension, diabetes, CAD, and active smoking.

^※^ BMI was not included in models

Abbreviations: BMI: body mass index, SBP: systolic blood pressure, LV: left ventricular, LVM: LV mass; LVMi: LV mass indexed to body surface area, LV M/V: LV mass-to-volume ratio, LVEDVi: indexed LV end-diastolic volume, E/e’: ratio of mitral peak velocity of early filling to early diastolic mitral annular velocity, e’: early diastolic mitral annular velocity, e’: early diastolic mitral annular velocity, E/e’: ratio of mitral peak velocity of early filling to early diastolic mitral annular velocity, GLS: global longitudinal strain; GCS: global circumferential strain, PALS: peak atrial longitudinal strain.

**Supplemental Materials**

**(Figures)**

**Supp. Figure 1.** Paired comparisons of key echocardiographic indices and deformational mechanics based on men, pre-menopausal, and post-menopausal women after propensity matching procedure.

**
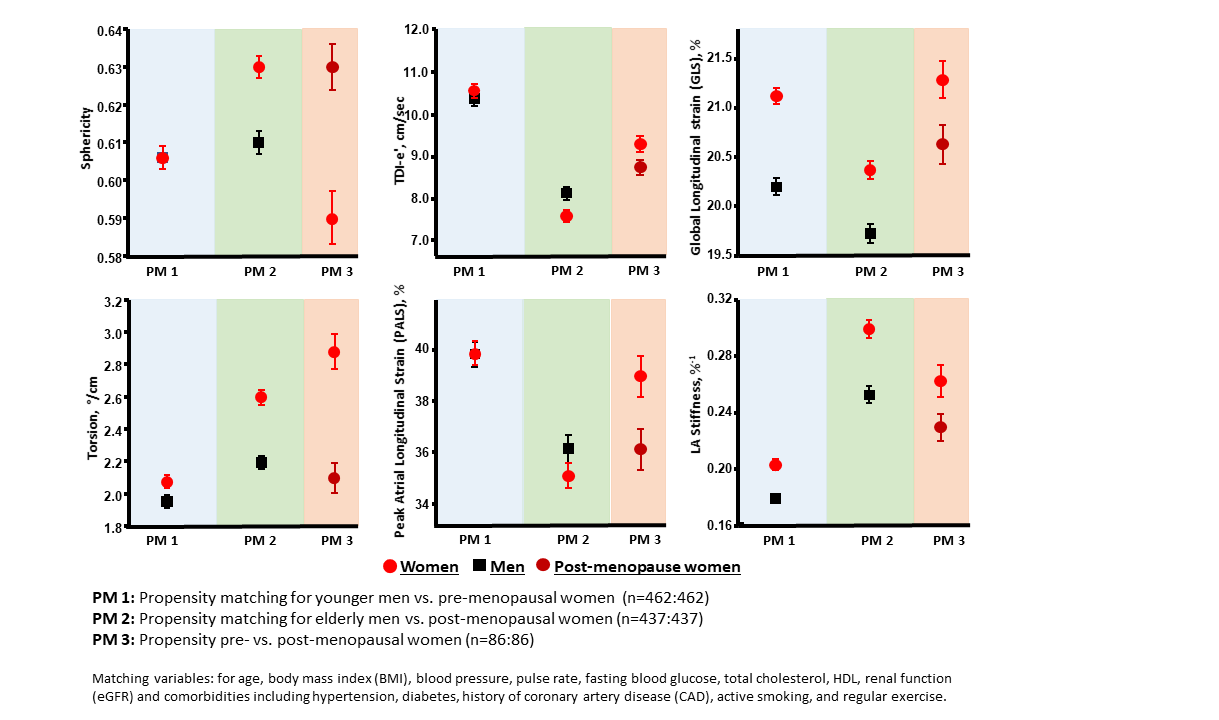
**

**Supp Figure 2.** Linear plotting illustrating associations of circulating Estradiol (E2) level with 4 key echocardiographic geometry and deformation indices including sphericity, GLS, torsion and PALS in women (pre- and post-menopause)

**

**

**Supp. Figure 3. Association between circulating estradiol (+10 ng/dL) level and measures of key cardiac structure and function among 320 women from an independent cohort**


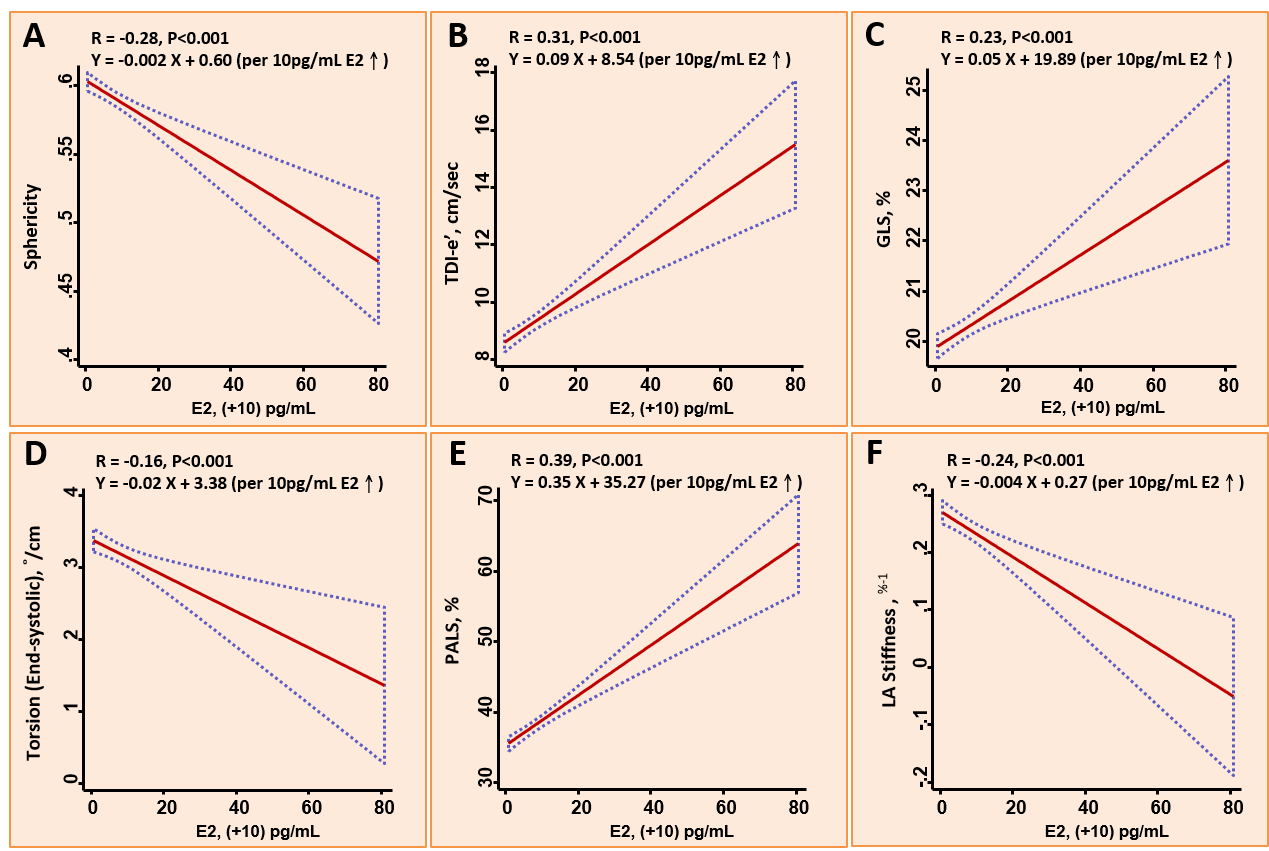


**Supp. Figure 4:** Kaplan-Meier survival curves for same echocardiographic geometry and deformation indices utilizing outcome-driven cut-offs based on outcomes of HF/all-cause death in post-menopausal women.


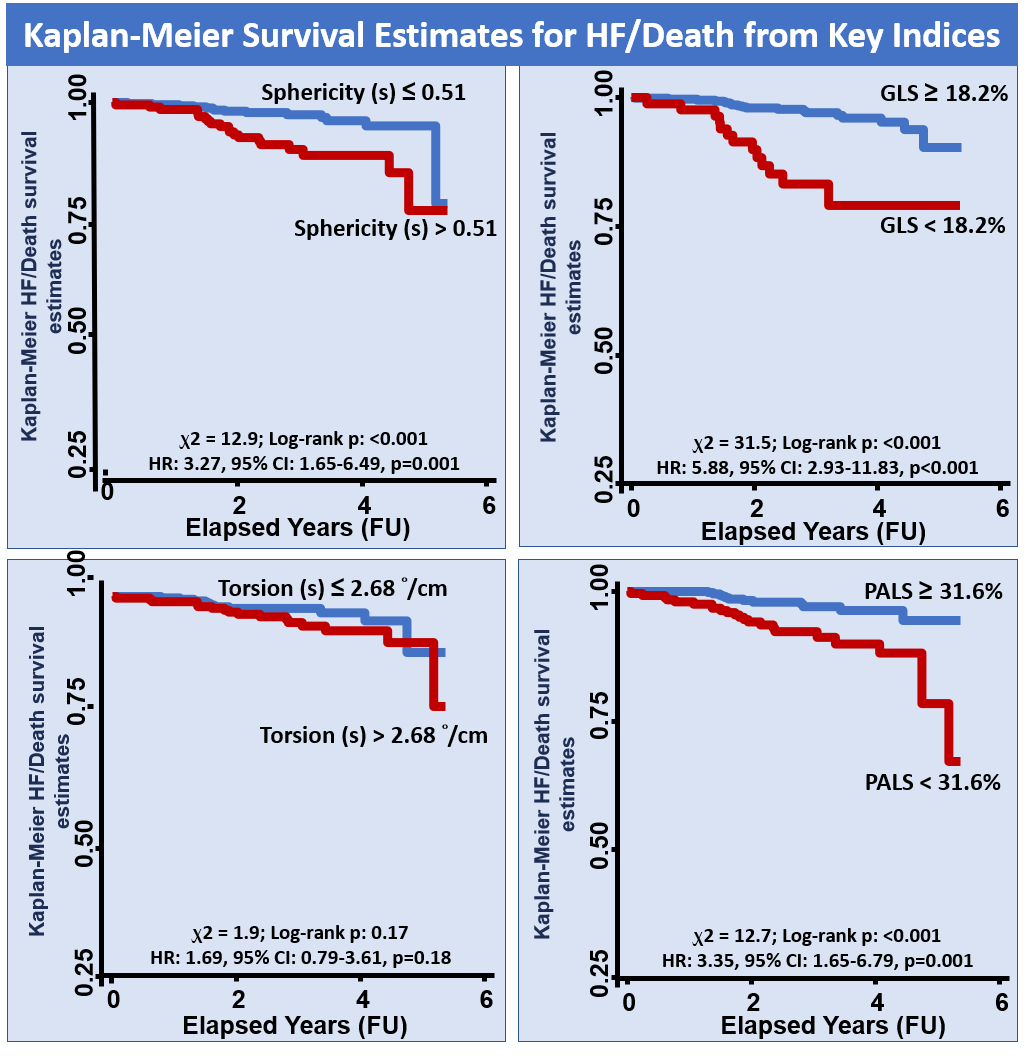

Supplement: Supplementary file 1 [file Data_Sheet_1.docx]
